# Podoplanin depletion in tonsil-derived mesenchymal stem cells induces cellular senescence via regulation of the p16^Ink4a^/Rb pathway

**DOI:** 10.1186/s12964-024-01705-8

**Published:** 2024-06-12

**Authors:** Ha Yeong Kim, Han Su Kim

**Affiliations:** Department of Otorhinolaryngology-Head and Neck Surgery, College of Medicine, Ewha Womans University, 1071 Anyangcheon-ro, Yangcheon-gu, Seoul, 07985 Republic of Korea

**Keywords:** Podoplanin, Tonsil-derived mesenchymal stem cells, Cellular senescence, p16, Transmembrane protein

## Abstract

**Background:**

Mesenchymal stem cells (MSCs) are widely used in the development of therapeutic tools in regenerative medicine. However, their quality decreases during in vitro expansion because of heterogeneity and acquired cellular senescence. We investigated the potential role of podoplanin (PDPN) in minimizing cellular senescence and maintaining the stemness of tonsil-derived MSCs (TMSCs).

**Methods:**

TMSCs were isolated from human tonsil tissues using an enzymatic method, expanded, and divided into two groups: early-passaged TMSCs, which were cultured for 3–7 passages, and late-passaged TMSCs, which were passaged more than 15 times. The TMSCs were evaluated for cellular senescence and MSC characteristics, and PDPN-positive and -negative cells were identified by fluorescence-activated cell sorting. In addition, MSC features were assessed in siRNA-mediated PDPN-depleted TMSCs.

**Results:**

TMSCs, when passaged more than 15 times and becoming senescent, exhibited reduced proliferative rates, telomere length, pluripotency marker (NANOG, OCT4, and SOX2) expression, and tri-lineage differentiation potential (adipogenesis, chondrogenesis, or osteogenesis) compared to cells passaged less than five times. Furthermore, PDPN protein levels significantly decreased in a passage-dependent manner. PDPN-positive cells maintained their stemness characteristics, such as MSC-specific surface antigen (CD14, CD34, CD45, CD73, CD90, and CD105) and pluripotency marker expression, and exhibited higher tri-lineage differentiation potential than PDPN-negative cells. SiRNA-mediated silencing of PDPN led to decreased cell-cycle progression, proliferation, and migration, indicating the significance of PDPN as a preliminary senescence-related factor. These reductions directly contributed to the induction of cellular senescence via p16^Ink4a^/Rb pathway activation.

**Conclusion:**

PDPN may serve as a novel biomarker to mitigate cellular senescence in the clinical application of MSCs.

**Graphical Abstract:**

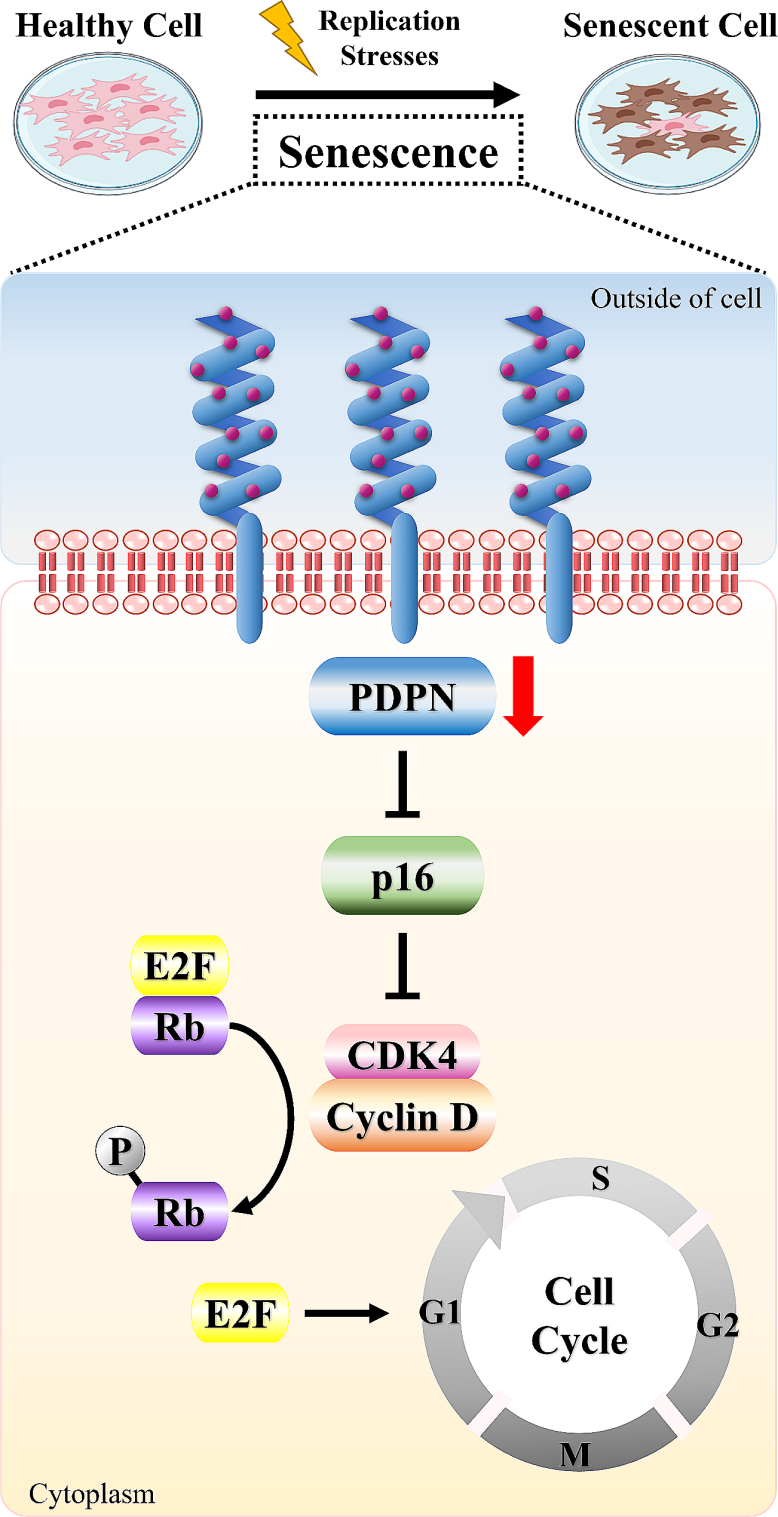

## Background

Stem cells have been applied as promising cell sources in clinical applications in regenerative medicine [1]. Particularly, adult stem cells, also known as mesenchymal stem cells (MSCs), have been widely investigated because they do not have the limitations in ethical issues or tumorigenicity associated with embryonic stem cells (ESCs) or induced pluripotent stem cells [2–5]. They can be acquired from diverse adult tissues, such as tonsils, fat, umbilical cord, and bone marrow [6, 7]. An increasing number of studies have reported the therapeutic effects of MSCs based on their properties of self-renewal, multipotency, and immunomodulatory actions that mediate the improvement of in vivo regeneration processes [8, 9]. Furthermore, MSCs release trophic factors that exert other therapeutic effects, such as pro- or anti-angiogenic effects. For example, exosomes released from mouse bone marrow-derived MSCs (BM-MSCs) significantly inhibit tumor progression and angiogenesis both in vitro and in vivo by downregulating vascular endothelial growth factor expression in 4T1 breast cancer cells [10]. Although MSCs have numerous advantages as therapeutic tools in regenerative medicine, several drawbacks prevent consistent quality under laboratory culture conditions. MSC populations can be expanded by passaging in vitro to achieve sufficient cell numbers; however, MSC proliferation decreases markedly with increasing passages, leading to cellular senescence [11].

Cellular senescence, a physiological process that results in the loss of replicative potential, was first discovered more than five decades ago by Leonard Hayflick and Paul Moorhead as an irreversible growth arrest of normal human fetal fibroblasts after long-term culture [12]. The general hallmarks identifying cellular senescence are the cessation of cell-cycle progression, relevant structural changes, e.g., in lysosomal compartments or the extracellular matrix, and metabolic adaptations, including increased reactive oxygen species production or dysfunctional mitochondria [13]. Moreover, the morphological and functional properties of MSCs can be altered by cellular senescence. The senescence of human BM-MSCs through extensive in-vitro expansion significantly impairs their phenotype and differentiation capability [14]. Human MSCs sourced from the umbilical cord (UC-MSCs) have the capacity for expansion over multiple passages; however, this often leads to genomic alterations, chromosomal anomalies, or changes in epigenetic characteristics [15]. Although the effects of cellular senescence have been reported in MSCs of various origins, the precise molecular mechanisms underlying these effects remain largely unclear.

The International Society for Cellular Therapy (ISCT) has reported definition criteria for MSCs; however, MSCs are highly heterogeneous because of various factors, including donor, tissue source, and cell-culture environment [16, 17]. Various surface phenotypic profiling approaches have been established to minimize the heterogenicity and senescence of MSCs. Positive selection for CD264 has been found to induce cellular senescence during serial passaging and reduce both proliferation and multilineage differentiation in human BM-MSCs as compared to CD264 negative selection [18]. In contrast, downregulation of CD146 results in low growth rates, multilineage differentiation, expression of pluripotency-related transcription factors (NANOG, OCT4, and SOX2), and high expression of senescence-related genes such as p53, p16, and p21 [19]. These findings suggest that the discovery of MSC senescence-associated biomarkers is critical for improving biological properties in vitro and therapeutic efficacy in vivo.

We previously found that tonsil-derived MSCs (TMSCs) have multipotent differentiation capacity to various lineage cells and a higher proliferation rate than other types of tissue-derived MSCs [20]. Therefore, we investigated the cellular mechanisms of MSC markers in regenerative medicine using TMSCs [21]. Recent studies have reported that podoplanin (PDPN), a small transmembrane glycoprotein, is found on the surface of certain MSCs, including TMSCs, and is related to aging, as evidenced by the decrease in PDPN expression in the papillary dermis of aging women, specifically those over the age of 40 years [22, 23]. PDPN is also known to regulate platelet activity, cellular migration, and proliferation [24]; however, most studies on these functional roles were confined to cells in lymphatic tissues or cancer. The few studies on stem cells have only reported their migratory ability; for example, high PDPN expression significantly enhanced the migration of UC-MSCs [25]. These reports suggest that studying the role of PDPN is important for understanding the aging and basic properties of MSCs. Therefore, in this study, we investigated how MSC properties are altered by replicative senescence using TMSCs and whether PDPN is involved in senescence-related changes and can be used as a biomarker of MSC stemness.

## Materials and methods

### Materials

High-glucose Dulbecco’s modified Eagle’s medium (DMEM-HG), low-glucose Dulbecco’s modified Eagle’s medium (DMEM-LG), and Dulbecco’s phosphate-buffered saline (DPBS) were purchased from Welgene Inc. (Gyeongsan, Korea). Collagenase type I, TRIzol reagent, dNTPs, Superscript™ III reverse transcriptase, Power SYBR™ Green PCR Master Mix, adipogenic, chondrogenic, and osteogenic differentiation medium, and Lipofectamine 2000 were purchased from Thermo Fisher Scientific (Waltham, MA, USA). DNase, 3-(4,5-dimethylthiazol-2-yl)-2,5-diphenyltetrazolium bromide (MTT) solution, and RNase A were purchased from Sigma-Aldrich (St. Louis, MO, USA). Ficoll-Paque was purchased from GE Healthcare (Piscataway, NJ, USA). Fetal bovine serum (FBS) was purchased from Young In Frontier (Seoul, Korea). A senescence-associated β-galactosidase (SA-β-gal) staining kit and antibodies against PDPN, phospho-p53-Ser^15^, p21, cyclin-dependent kinase 4 (CDK4), retinoblastoma (Rb), and phospho-Rb-Ser^807/811^ were purchased from Cell Signaling Technology (Boston, MA, USA). A DNeasy Blood & Tissue kit was purchased from Qiagen (Hilden, Germany). Flow-cytometry antibodies against CD14, CD34, CD73, CD45, CD90, CD105, and PDPN, antibody against cyclin D1, and propidium iodide (PI) were purchased from BD Biosciences (San Jose, CA, USA). The cell strainer was purchased from Corning Inc. (Corning, NY, USA). Antibodies against p53 and control small interfering (si) RNA (siCON) were purchased from Santa Cruz Biotechnology (Santa Cruz, CA, USA). Antibody against p16 was purchased from Abcam (Cambridge, UK). Antibody against glyceraldehyde 3-phosphate dehydrogenase (GAPDH) was purchased from AbFrontier (Seoul, Korea). PDPN siRNA (siPDPN) was purchased from Bioneer Inc. (Oakland, CA, USA).

### Isolation and expansion of TMSCs

TMSCs were isolated and expanded following a previously reported protocol approved by the Institutional Review Board (EUMC-2019-08-030) of Ewha Womans University Mokdong Hospital, Korea [20]. Written informed consent was obtained from the legal guardians of patients undergoing tonsillectomy. Briefly, tonsil tissues collected from donors were mechanically cut into small pieces using scissors and enzymatically digested in DMEM-HG supplemented with 210 U/mL collagenase type I and 10 mg/mL DNase at 37 °C for 30 min. The digested cells were filtered and centrifuged with Ficoll-Paque ready-to-use density gradient media to obtain mononuclear cells, which were plated and incubated for 48 h. Adherent cells, termed TMSCs, were used in the experiments.

For experiments, TMSCs were cultured and expanded in DMEM-LG supplemented with 10% FBS, 100 U/mL penicillin-streptomycin, and 0.25 µg/mL amphotericin B in a humidified CO_2_ incubator at 37 °C. For comparative experiments, cells were divided into two groups based on passage number ranges; early-passaged TMSCs were passaged 3–7 times, and late-passaged TMSCs were passaged > 15 times.

### Measurement of doubling time and cell size

TMSCs (5 × 10^5^) were seeded in a 100-mm cell culture dish and subcultured every 72 or 96 h. Viable cells stained with trypan blue were counted using a hemocytometer. We determined the initial cell number and final TMSCs count at each passage and calculated doubling time using the following formula:

$$\rm{Doubling\ time\ (h)} = \left[\left\{\left(\text{T}-{\text{T}}_{0}\right)\left({\text{log}}_{0})/\left(\text{log}\text{N}-\text{log}{\text{N}}_{0}\right)\right.\right\}\right]$$

where T is the time and N is the cell count.

In the separative experiments, TMSCs were seeded in culture plates to the indicated passages and observed under an optical microscope (BX51; Olympus, Tokyo, Japan) after 24 h. Cell length and extension width were measured in at least five random fields using the ImageJ software (National Institutes of Health, Bethesda, MD, USA).

### SA-β-gal assay

SA-β-gal activity was detected in early- and late-passaged cells using an SA-β-gal staining kit according to the manufacturer’s instructions. In brief, TMSCs were fixed in a fixative solution at room temperature for 15 min and then washed twice with DPBS. The fixed cells were incubated with β-gal staining solution (pH 6.0) in a CO_2_-free dry incubator at 37 °C for 24 h. Senescent cells were stained blue and observed under an optical microscope.

### Reverse transcription-polymerase chain reaction (RT-PCR)

Total RNA was extracted from TMSCs using TRIzol reagent and used to synthesize cDNA using dNTPs, oligo-dT primers, and Superscript™ III reverse transcriptase according to the manufacturer’s instructions. The reaction conditions of PCR amplification were one cycle at 94 °C for 2 min, 36 cycles at 94 °C for 30 s, 56 °C for 30 s, 72 °C for 1 min, and 72 °C for 5 min. The primer sequences are listed in Table 1.

**Table 1:** Human gene-specific primers used for RT-PCR

Target gene	Primer sequence (5′→3′)
*NANOG*	F: 5′- ATG GTG TGA CGC AGG GAT G-3′ R: 5′-TGG TTG CTC CAG GTT GAA TTG-3′
*OCT4*	F: 5′-CTT GAA TCC CGA ATG GAA AGG G-3′ R: 5′-GTG TAT ATC CCA GGG TGA TCC TC-3′
*SOX2*	F: 5′-AAC CAA GAC GCT CAT GAA GAA G-3′ R: 5′-CAT GTG CGC GTA ACT GTC C-3′
*PDPN*	F: 5′-AGG TGC CGA AGA TGA TGT GG-3′ R: 5′-AGT TGG CAG ATC CTC GAT GC-3′
*GAPDH*	F: 5′-GGA GCG AGA TCC CTC CAA AAT-3′ R: 5′-GGC TGT TGT CAT ACT TCT CAT GG-3′
Telomere	Telg: 5′-GGA GCG AGA TCC CTC CAA AAT-3′ Telc: 5′-GGA GCG AGA TCC CTC CAA AAT-3′
*36B4*	F: 5′-CAG CAA GTG GGA AGG TGT AAT CC-3′ R: 5′-CCC ATT CTA TCA TCA ACG GGT ACA A-3′

### Measurement of telomere length

Genomic DNA was extracted from TMSCs using a DNeasy Blood & Tissue kit according to the manufacturer’s instructions. The extracted genomic DNA was used to determine relative telomere length, which is the ratio of telomeres to single-copy genes (T/S ratio), as previously described [26]. In brief, genomic DNA (200 ng/µL) was subjected to PCR using Power SYBR™ Green PCR Master Mix and a telomere primer pair (telg and telc) or a 36B4 primer pair for single-copy gene amplification on a QuantStudio 3 real-time PCR system (Thermo Fisher Scientific).

### Flow-cytometric analysis

The immunophenotypes of TMSCs were examined using flow cytometry. TMSCs were collected as single cells and stained with phycoerythrin (PE)- or fluorescein isothiocyanate (FITC)-conjugated anti-human antibodies against CD14, CD34, CD73, CD45, CD90, CD105, and PDPN at 4 °C for 30 min. The stained cells were analyzed using an ACEA NovoCyte flow cytometer (Agilent Technologies Inc., Santa Clara, CA, USA).

### Fluorescence-activated cell sorting (FACS)

TMSCs were prepared as single cells and labeled with PE-conjugated anti-human PDPN. The cells were washed twice with cell sorting buffer containing FBS and filtered using a 70-µm cell strainer. The filtered single cells were sorted into positive or negative cells on an S3e™ cell sorter (Bio-Rad Laboratories Inc., Hercules, CA, USA). After sorting, the cells were seeded and cultured for further experiments.

### Tri-lineage differentiation of TMSCs

To examine the level of tri-lineage differentiation, TMSCs were cultured in commercially available adipogenic, chondrogenic, or osteogenic differentiation medium for 2 weeks. The differentiation medium was changed every 3 or 4 days. Differentiated TMSCs were fixed with 4% paraformaldehyde for 30 min and washed with DPBS. Oil red O, Alcian blue, and Alizarin red S staining were used to assess and validate the adipogenic, chondrogenic, and osteogenic differentiation capacity of cells, respectively. After staining for 30 min, the cells were washed twice with DPBS and observed under a microscope. At least three random images were converted into an RGB stack to quantify the level of differentiation using ImageJ.

### Western blot analysis

Proteins were extracted from TMSCs using a lysis buffer containing 20 mM Tris-HCl (pH 7.5), 150 mM NaCl, 1% Triton X-100, 1 mM EDTA, and 1 mM EGTA. Proteins (20 µg) were separated by sodium dodecyl sulfate-polyacrylamide gel electrophoresis and transferred onto nitrocellulose membranes using the Trans-Blot Turbo system (Bio-Rad Laboratories Inc.). The membranes were blocked with 5% skim milk and probed with primary antibodies against the following proteins: PDPN, phospho-p53-Ser^15^, p21, CDK4, Rb, phospho-Rb-Ser^807/811^, p53, p16, cyclin D1, and GAPDH. The protein blots were developed on a luminescent image analyzer (Cytiva, Marlborough, MA, USA) using a chemiluminescent reagent (Amersham, Buckinghamshire, UK).

### Cell viability/proliferation assays

After cell sorting into PDPN-positive (PDPN^+^) and PDPN-negative (PDPN^−^) cells, cell viability/proliferation assays were carried out as described previously [27], with a minor modification. In brief, sorted cells were plated in 24-well plates at a density of 1 × 10^4^ cells per well and incubated with MTT solution in a humidified CO_2_ incubator at 37 °C for 2 h at the indicated times (0, 24, 48, or 72 h). Following the incubation period, the purple formazan crystals were solubilized with dimethyl sulfoxide. The absorbance at 540 nm was measured and compared with the baseline value at time 0.

### Cell-cycle analysis

TMSCs (1 × 10^5^) were prepared as single cells and fixed with a 70% ethanol solution at 4 °C for 1 h. The fixed cells were washed with cold DPBS supplemented with 2% FBS and then stained with PI/RNase A solution for 30 min. For each sample, the cell-cycle distribution was determined using the ACEA NovoCyte flow cytometer.

### In-vitro migration assay

TMSCs were plated in a six-well plate at a density of 3 × 10^5^ cells per well. After 24 h, cells that had reached 80–90% confluence were treated with 10 µg/mL mitomycin C in a humidified CO_2_ incubator at 37 °C for 2 h. The cells were gently washed with DPBS and supplied with a culture medium. A linear wound was created by scratching with a 200-µL pipette tip. At 0 and 24 h, the wound was imaged using a microscope. Wound closure was determined as the percentage of the residual gap relative to the initial wound area.

### Transfection

TMSCs at 80% confluence were transfected with 100 nM siCON or siPDPN using Lipofectamine 2000 according to the manufacturer’s instructions. After transfection, the cells were incubated for 24 h and then used for further experiments.

### Statistical analysis

Data are expressed as mean ± standard deviation (SD). Statistical significance was assessed using Student’s *t*-test or one-way analysis of variance. Statistical significance was set at *p* < 0.05. To ensure the reliability of the findings, all experiments were conducted at least three times.

## Results

### Long-term expansion induces cellular senescence of TMSCs

**Fig. 1:**
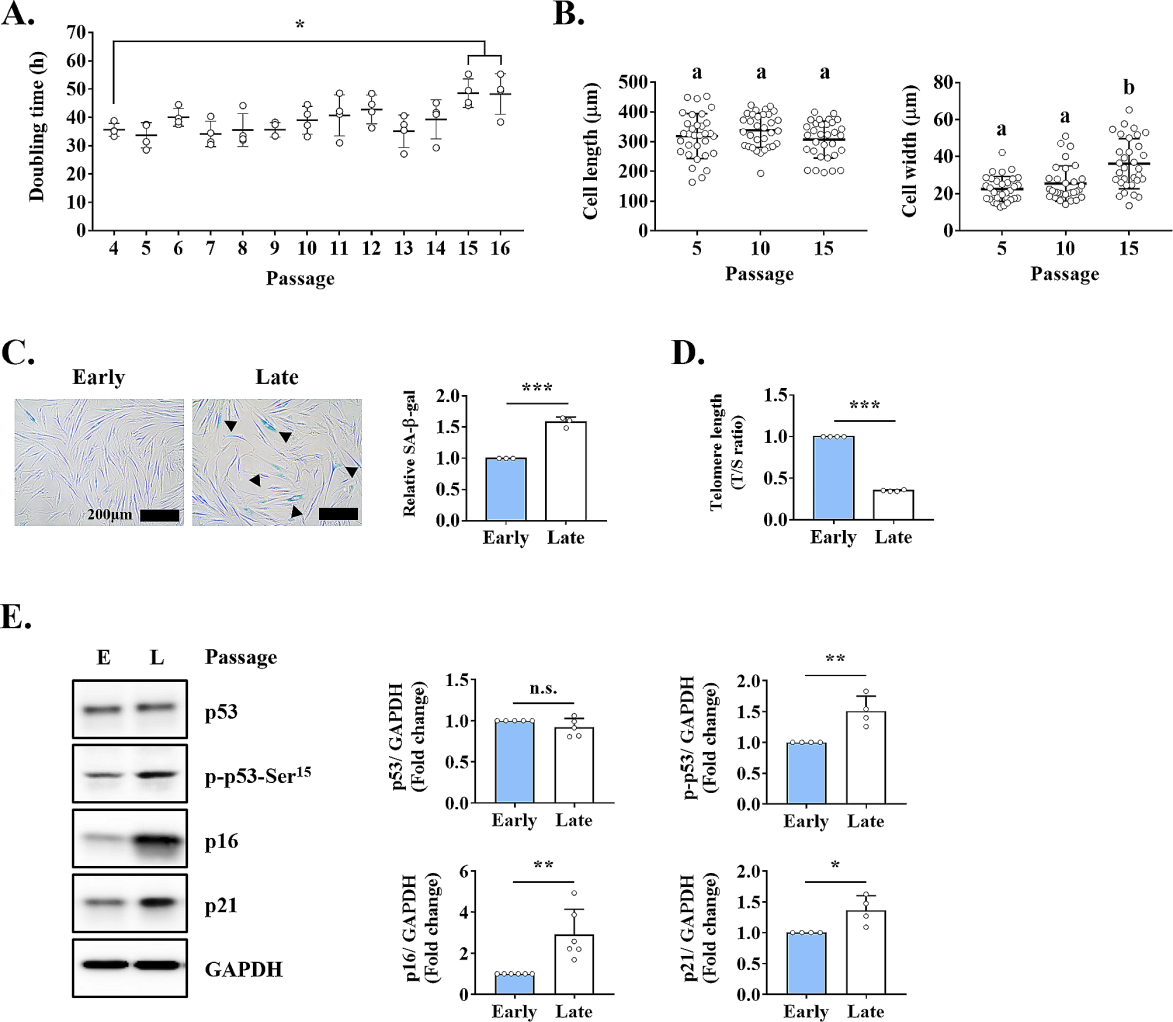
Characterization of replicative senescence in TMSCs. **(A)** Doubling time, indicative of cell growth, of TMSCs in continuous culture. **(B)** Cell length and width in function of the number of passages. **(C)** SA-β-gal assay to determine senescent cells in early- and late-passaged cell groups. Blue-stained senescent cells (black arrow) and the relative intensity of SA-β-gal staining were quantified in at least three random images using the ImageJ software. Early-passaged TMSCs were passaged 3–7 times and late-passaged TMSCs were passaged > 15 times. Scale bar = 200 μm. **(D)** Telomere length as determined by the ratio of telomere to single-copy gene expression (T/S ratio). **(E)** Protein levels of cellular senescence-associated markers (p53, p-p53-Ser^15^, p16, and p21) as measured using western blotting. Results are representative of at least three independent experimental repeats. Data are mean ± SD. *p < 0.05, **p < 0.01, and ***p < 0.001. n.s., not significant. Different letters indicate significant differences among experimental groups (*p < 0.05). Abbreviations: Early or E, early-passaged TMSCs; Late or L, late-passaged TMSCs

We evaluated the growth rate and morphological changes of TMSCs according to the number of passages during cell culture. TMSCs obtained from four donors of the same age group were serially cultured, and the doubling time from passages 4 to 16 was calculated. The average doubling time before passage 14 was 37.42 ± 5.30 h, but it significantly increased to 48.41 ± 5.70 h with additional passages (Fig. 1A). Morphologic changes were assessed by measuring cell length and width. Cell length did not significantly change up to passage 15; it was 318.67 ± 75.30 μm at passage 5, 338.46 ± 57.13 μm at passage 10, and 307.28 ± 62.57 μm at passage 15. However, cell width increased with passaging (22.61 ± 6.73 μm at passage 5, 25.52 ± 9.50 μm at passage 10, and 36.21 ± 13.50 μm at passage 15), and cells were significantly wider at passage 15 than at passage 5 (Fig. 1B).

Based on the data Fig. 1A and B, we divided TMSCs into two groups to confirm the passage-dependent senescence of TMSCs: early-passaged TMSCs, cultured for 3–7 passages, and late-passaged TMSCs, cultured for > 15 passages. SA-β-gal staining showed a significantly higher degree of cellular senescence in late-passaged than in early-passaged TMSCs (Fig. 1C). Telomeres are noncoding, double-stranded nucleotide repeats (TTAGGG)n that shorten with each cell division [28]. This progressive telomere shortening leads to cellular senescence [29]. The T/S ratio significantly decreased over time, corroborating the association between telomere shortening and replicative senescence (Fig. 1D).

The most defining hallmark of cellular senescence is cell cycle arrest, which is governed by two major pathways: p16^Ink4a^/Rb and p53/p21^CIP1^. We found that late-passaged TMSCs had significantly higher levels of markers such as p16, p21, and phospho-p53 than early-passaged TMSCs (Fig. 1E), suggesting that TMSCs cultured for > 15 passages become senescent as a result of consecutive passaging.

### MSC characteristics change through replicative senescence

**Fig. 2:**
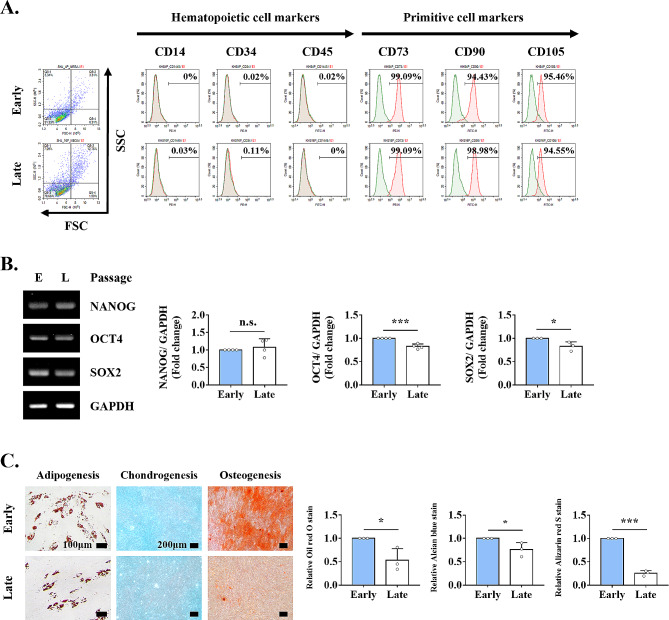
Effects of senescence on MSC characteristics. Early- and late-passaged TMSCs were evaluated for MSC characteristics based on the ISCT minimum criteria. **(A)** Flow cytometry-based forward scatter and side scatter values were used to assess cell size and granularity, as well as MSC-specific surface marker expression, including hematopoietic surface markers (CD14, CD34, and CD45) and primitive cell markers (CD73, CD90, and CD105). **(B)** Gene expression of pluripotency-related transcription factors (NANOG, OCT4, and SOX2) as determined by RT-PCR. **(C)** Relative levels of multilineage differentiation as evaluated by measuring the accumulation of lipid droplets stained with Oil red O (adipogenesis), glycosaminoglycans stained with Alcian blue (chondrogenesis), and calcium deposition stained with Alizarin red S (osteogenesis). The results are representative of at least three independent experimental repeats. Data are mean ± SD. *p < 0.05 and ***p < 0.001. n.s., not significant. Abbreviations: Early or E, early-passaged TMSCs; Late or L, late-passaged TMSCs

We next investigated whether MSC characteristics, such as the immunological surface phenotype, pluripotency marker expression, and tri-lineage differentiation capacity, were altered through replicative senescence. Immunological characteristics were analyzed using flow cytometry, and forward and side scatter values for assessing cell size and granularity were increased in late- compared to early-passaged TMSCs (Fig. 2A). However, stem cell-surface phenotypic marker expression as suggested in the ISCT minimum criteria, including negative expression for hematopoietic surface markers (CD14, CD34, and CD45) and positive expression for primitive cell markers (CD73, CD90, and CD105), did not differ between the two groups.

MSCs express transcription factors that maintain pluripotency in ESCs (NANOG, OCT4, and SOX2). RT-PCR showed that the expression of OCT4 and SOX2, but not that of NANOG, was significantly decreased in late-passaged TMSCs undergoing replicative senescence (Fig. 2B).

We next investigated the effects of replicative senescence on tri-lineage differentiation capacity (adipogenesis, chondrogenesis, and osteogenesis). The mesodermal differentiation potential was significantly decreased in replicative senescent cells compared to early-passaged TMSCs, as evidenced by reduced Oil red O, Alcian blue, and Alizarin red S staining (Fig. 2C).

### PDPN expression decreases during senescence in TMSCs

**Fig. 3:**
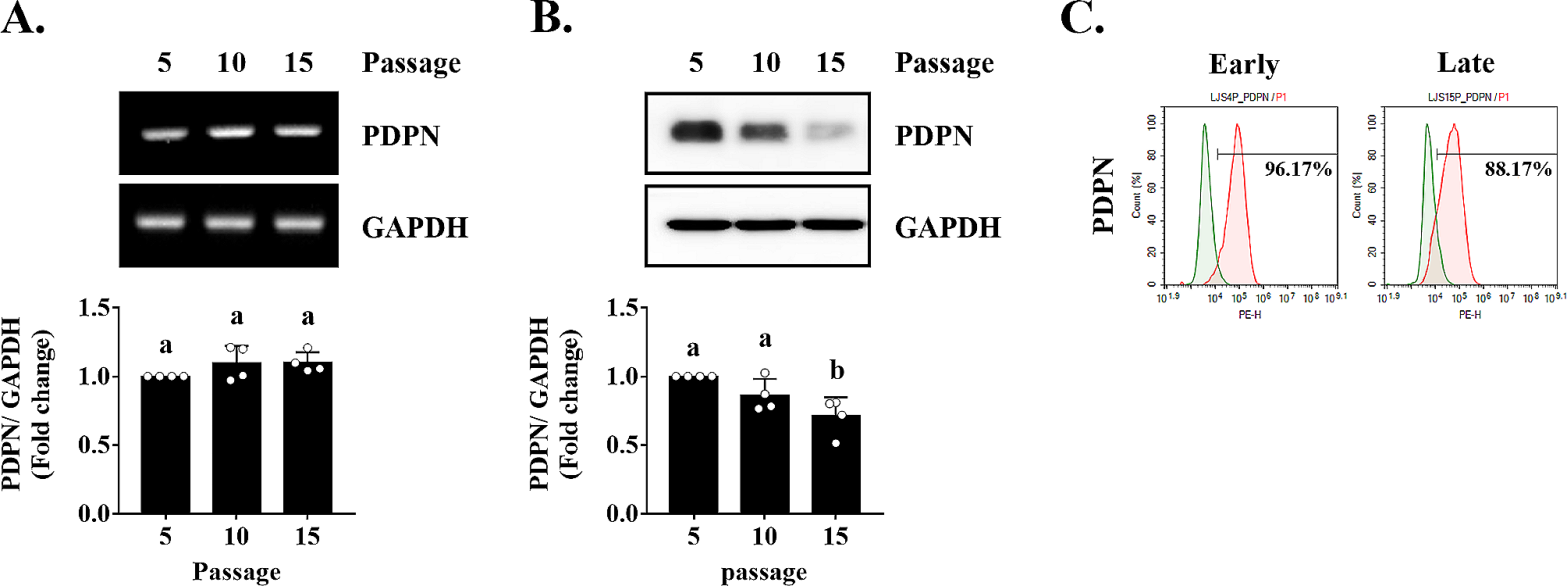
PDPN expression during replicative senescence. **(A)** mRNA and **(B)** protein levels of PDPN and GAPDH were measured using RT-PCR and western blotting, respectively. **(C)** Immunophenotypic analysis of PDPN in early- and late-passaged cells as assessed by flow cytometry. The results are representative of at least three independent experimental repeats. Data are mean ± SD. Different letters indicate significant differences among experimental groups (*p < 0.05). Abbreviations: Earl, early-passaged TMSCs; Late, late-passaged TMSCs

We measured PDPN gene and protein expression during serial culture of TMSCs. PDPN mRNA levels did not differ up to passage 15, but PDPN protein levels decreased in a passage-dependent manner (Fig. 3A, B). In particular, there was a significant decrease at passage 15 compared to passage 5. Flow-cytometric analysis revealed that transmembranal PDPN expression was decreased by approximately 10% in the late-passaged TMSC group (Fig. 3C). These results indicated that the PDPN expression decreases through replicative senescence in TMSCs.

### PDPN sorting affects the MSC characteristics of TMSCs

**Fig. 4:**
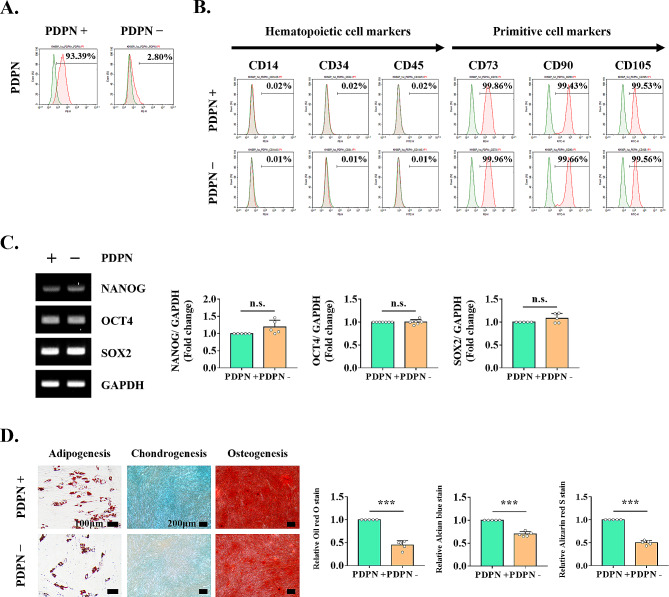
Identification of MSC characteristics in PDPN^+^ and PDPN^−^ cells sorted by FACS. **(A)** Immunophenotypic analysis of PDPN on PDPN^+^ cells and PDPN^−^ cells using flow cytometry. **(B)** Flow cytometry-based analysis of MSC-specific surface marker expression. **(C)** Pluripotency markers and **(D)** multilineage differentiation potential of PDPN^+^ cells and PDPN^−^ cells. The results are representative of at least three independent experimental repeats. Data are mean ± SD. ***p < 0.001. n.s., not significant. Abbreviations: PDPN^+^, PDPN-positive cells; PDPN^−^, PDPN-negative cells

We next investigated whether the expression of PDPN in TMSCs would affect their MSC characteristics. PDPN is a membrane protein expressed on the cell surface and can be used for positive selection using cell separation technology, such as FACS. Therefore, we performed FACS analysis to isolate cells with PDPN-positive or -negative expression. After isolation, PDPN^+^ and PDPN^−^ TMSCs were identified at ratios of 93.39% and 2.80%, respectively (Fig. 4A). Stem cell-surface phenotypic markers and pluripotency-related transcription factors showed similar expression levels in PDPN^+^ and PDPN^−^ cells (Fig. 4B, C). However, the tri-lineage differentiation potential of PDPN^+^ TMSCs was significantly higher than that of PDPN^−^ TMSCs, as evidenced by increased accumulation of lipid droplets stained with Oil red O (adipogenesis), glycosaminoglycans stained with Alcian blue (chondrogenesis), and calcium deposition stained with Alizarin red S (osteogenesis) (Fig. 4D).

### PDPN depletion induces cellular senescence by activating the p16^Ink4a^/Rb signaling pathway

**Fig. 5:**
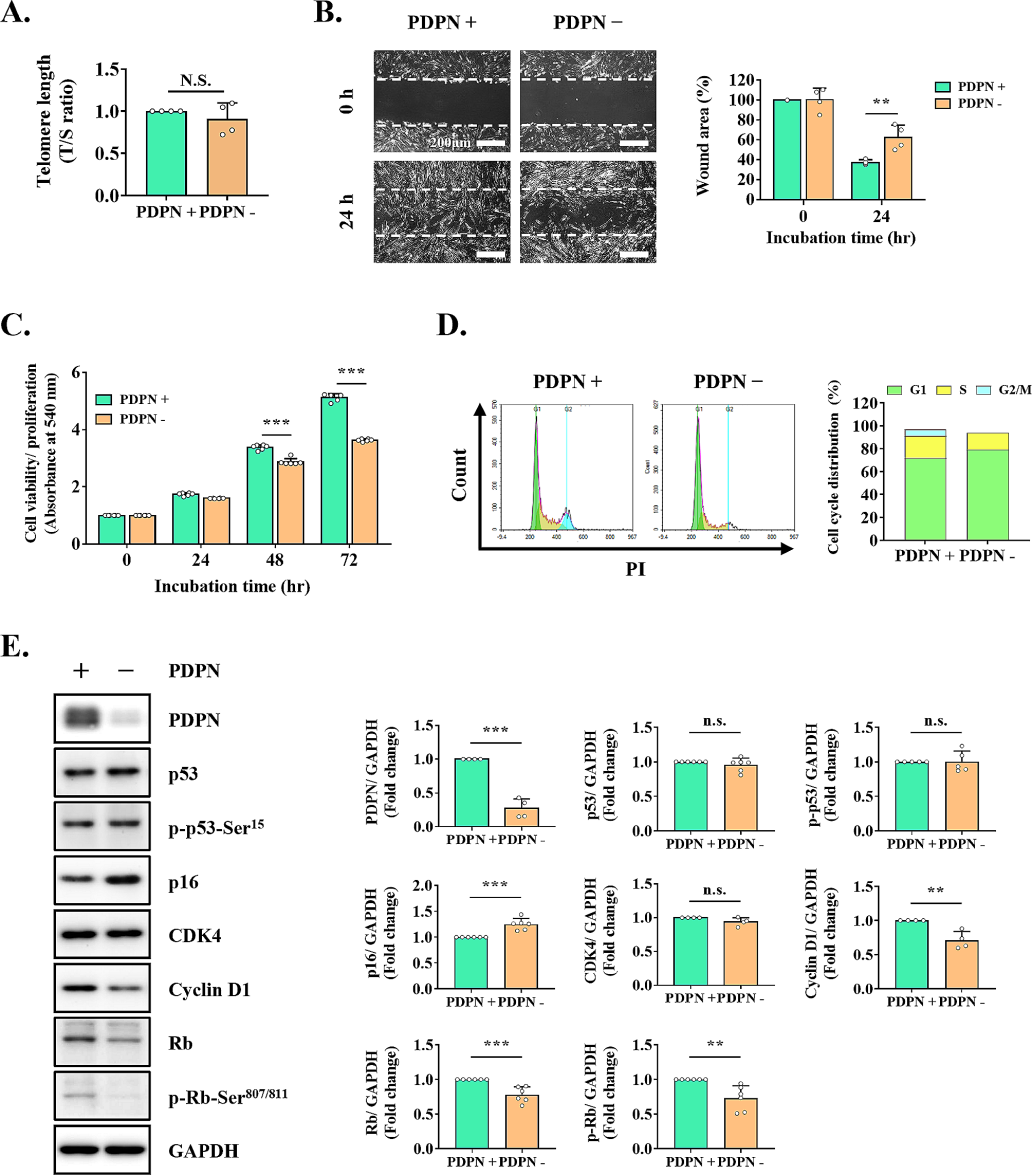
Effects of cellular senescence-related mechanism after sorting PDPN^+^ cells by FACS. **(A)** Telomere length in PDPN^+^ and PDPN^−^ cells as assessed by measuring the T/S ratio. **(B)** Cell migration as analyzed by in vitro wound healing assays. The wound closure rate was calculated as the percentage of remaining wound area compared to the initial wound area. Scale bar = 200 μm. **(C)** Difference in cell proliferation capacity between PDPN^+^ and PDPN^−^ cells for up to 72 h. **(D)** Cell-cycle analysis of PDPN^+^ and PDPN^−^ cells using flow cytometry and PI staining. **(E)** Protein levels of PDPN and senescence- and cell cycle-associated markers (p53, p-p53-Ser^15^, p16, CDK4, cyclin D1, Rb, and p-Rb-Ser^807/811^) as measured by western blotting. The results are representative of at least three independent experimental repeats. Data are mean ± SD. **p < 0.01 and ***p < 0.001. n.s., not significant. *Abbreviations* PDPN^+^, PDPN-positive cells; PDPN^−^, PDPN-negative cells

To determine the effects of PDPN on the senescence of TMSCs, we explored the association of PDPN with senescence features, such as telomere shortening, cell migration, proliferation capacity, and cell-cycle arrest. Initially, we investigated whether sorting PDPN^+^ TMSCs would affect their telomere length. However, we did not observe a significant difference in telomere length between PDPN^+^ and PDPN^−^ cells (Fig. 5A). Cellular senescence can interfere with mechanisms associated with wound healing, including proliferation and migration. Therefore, a wound healing assay was used to evaluate cell migration. PDPN^+^ cells had significantly higher wound closure capacity than PDPN^−^ cells (wound area, 37.28 ± 2.66% vs. 62.63 ± 12.16%) (Fig. 5B). As shown in Fig. 5C, PDPN^+^ cells had a significantly higher proliferation potency after 48 h in culture than PDPN^−^ cells.

To investigate cell-cycle arrest, we examined the PDPN signaling pathway through cell-cycle analysis using flow cytometry, along with the expression of senescence- or cell cycle-related proteins. As shown in Fig. 5D, the percentages of cells in the G1, S, and G2/M phases were 72.72%, 19.87%, and 6.9%, respectively, for PDPN^+^ TMSCs, and 79.73%, 15.96%, and 0.28%, respectively, for PDPN^−^ TMSCs. These results indicated that PDPN^−^ TMSCs demonstrated an increased induction of cell-cycle arrest in the G1 phase compared to PDPN^+^ TMSCs (Fig. 5D). Further validation revealed that the level of cyclin D1, a representative regulatory protein of the G1 phase, was significantly lower in PDPN^−^ TMSCs. However, the level of CDK4, another marker associated with the G1 phase, did not significantly differ (Fig. 5E). We then investigated the signaling pathway involved in G1 arrest. The transition from the G1 to the S phase is controlled by the p16^Ink4a^/Rb signaling pathway [30]. As expected, p16 protein expression was significantly increased in PDPN^−^ TMSCs, whereas its downstream mediators, Rb and phospho-Rb, were significantly downregulated. However, the expression levels of p53 and phospho-p53, which are other modulators of cell-cycle arrest, did not show significant differences (Fig. 5E).

### PDPN knockdown promotes p16-dependent cellular senescence

**Fig. 6:**
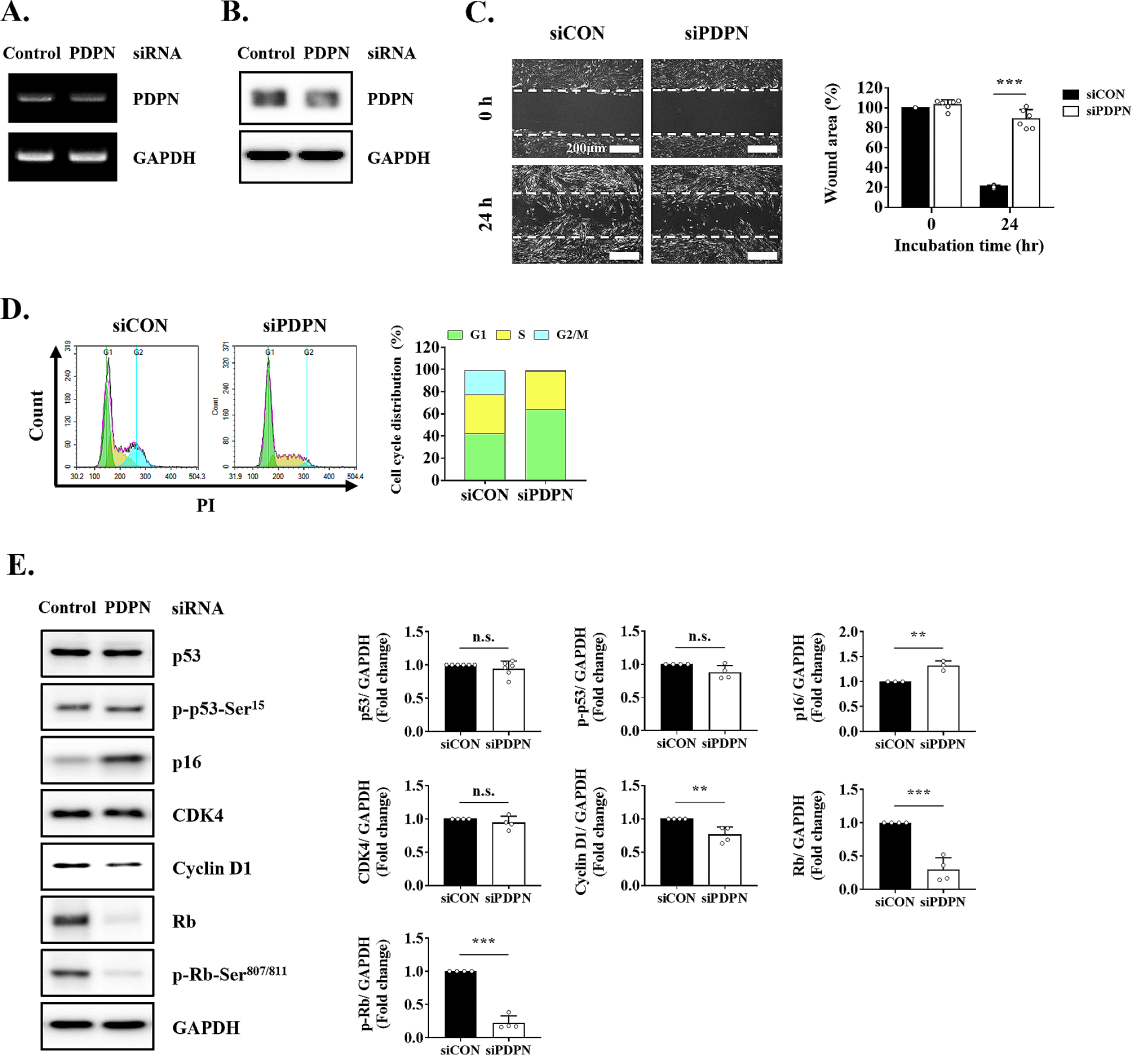
Assessment of the cellular senescence-related mechanism after transfection with PDPN siRNA. After siRNA transfection, **(A)** mRNA and **(B)** protein levels of PDPN and GAPDH were measured using RT-PCR and western blotting, respectively. **(C)** Cell migration as measured as in vitro wound healing assays. Scale bar = 200 μm. **(D)** Cell-cycle analysis in cells transfected with PDPN siRNA using flow cytometry and PI staining. **(E)** Protein levels of senescence- and cell cycle-associated markers (p53, p-p53-Ser^15^, p16, CDK4, cyclin D1, Rb, and p-Rb-Ser^807/811^) as measured by western blotting. The results are representative of at least three independent experimental repeats. Data are mean ± SD. **p < 0.01 and ***p < 0.001. n.s., not significant

The role of PDPN in cellular senescence was confirmed by silencing its expression using siRNA. Ectopic transfection with PDPN siRNA successfully reduced both mRNA and protein levels of PDPN (Fig. 6A, B). The residual wound area was higher in the siPDPN group than in the control group (Fig. 6C). As expected, siPDPN completely inhibited cell-cycle progression at the G1 phase as compared to siCON (Fig. 6D**)**. Moreover, PDPN knockdown significantly altered p16^Ink4a^/Rb signaling, including the induction of p16 protein expression and the suppression of cyclin D1, Rb, and phospho-Rb protein levels (Fig. 6E). Taken together, these results suggested that loss of PDPN leads to the induction of cellular senescence, resulting in the downregulation of cell-cycle progression as well as proliferation and migration in TMSCs.

Schematic illustration of the mechanisms involved in the cellular senescence of TMSCs through the PDPN-mediated p16^Ink4a^/Rb pathway

## Discussion

MSC-based therapies have rapidly expanded to target various diseases; however, there are concerns regarding their functional capacity declining with aging, suggesting that high efficacy is essential. In this study, we investigated changes in the phenotypic and functional characteristics of TMSCs undergoing senescence and the involvement of PDPN in this process.

Our study revealed the regulatory role of PDPN in delaying cellular senescence via inhibition of cell-cycle progression, proliferation, and migration in TMSCs. PDPN, also known as E11 antigen, is a unique membrane protein composed of a highly O-glycosylated extracellular domain, a single membrane-spanning region, and a short cytoplasmic tail [31]. It is widely expressed in various tissues and cell types, suggesting an important role in embryonic development; however, little is known about its physiological functions. PDPN reportedly serves as a regulator of cell proliferation and migration in numerous cell types [24]. However, the role of PDPN in MSCs remains to be fully elucidated as previous reports focused only on the migratory ability of MSCs. We observed that low PDPN expression in TMSCs inhibited cell migration and proliferation, suggesting a mechanistic link with cell-cycle arrest. G1 phase transition reportedly is associated with the regulation of cell migration and proliferation [32]. For example, in NCI-H23 human lung cancer cells, caffeine inhibited proliferation, migration, and invasion by enhancing G1 cell-cycle arrest [33]. Although PDPN knockdown has been reported to induce cell-cycle arrest in the G2/M phase through activation of the p53 pathway [34, 35], we found that inducing low levels of PDPN through cell sorting or gene silencing led to cell-cycle arrest in the G1 phase. This is the first study to demonstrate an association between PDPN and G1 cell-cycle arrest. In general, G1 or G2 cell-cycle arrest is sufficient for the induction of cellular senescence and is a well-known defining feature of senescent cells [36]. Particularly, cellular senescence upon this irreversible growth arrest state is predominantly regulated by the p53/p21^CIP1^ and p16^Ink4a^/Rb signaling pathways, both of which are closely interlinked [30, 37, 38]. However, we found that PDPN regulates the cell cycle via p53-independent p16 activation. In line herewith, Jacobs et al. reported that p16^Ink4a^ activation significantly induced telomere-directed senescence in human fibroblasts through a p53-independent response [39].

Ectopic expression of p16, a cyclin-dependent kinase inhibitor, triggers cellular senescence by controlling cell-cycle progression from the G1 to the S phase [40, 41]. In this process, p16 inhibits the formation of cyclin D-CDK4/6 complexes, thereby preventing the phosphorylation of Rb, which interacts with E2F family transcription factors [30]. Consistent with these previous findings, we found that PDPN knockdown increased p16 expression, which inhibited not only the phosphorylation but also the protein expression of Rb. The downregulation of Rb expression may have been mediated by upregulated p16 because p16 has multiple biological functions, including the transcriptional repression of several genes, including Rb [42]. Furthermore, we observed a significant decrease in cyclin D1 expression, an upstream regulatory factor of Rb. Cyclins and CDKs, which are essential in each cell-cycle phase, form complexes, notably the cyclin D1-CDK4 complex, which is specifically associated with G1 arrest [43, 44]. Interestingly, our experiments showed that CDK4 expression remained unchanged, suggesting potential cyclin D1-independent functions of CDKs in cell-cycle processes [45, 46]. Cyclin D1 acts as a regulatory protein that activates the cyclin-CDK complex, which remains relatively inactive in the absence of cyclins [47]. Overall, our study findings suggest that low PDPN expression in TMSCs can induce cellular senescence by activating the p16^Ink4a^/Rb pathway, leading to the inhibition of cell migration and proliferation, which is closely associated with G1 cell-cycle arrest. Although the detailed mode of action regarding how PDPN activates the p16^Ink4a^/Rb pathway has not yet been fully elucidated, previous studies suggested that PDPN interacts with the standard isoform of CD44 to activate the phosphatidylinositol 3-kinase (PI3K)/Akt signaling pathway [48, 49], which regulates MSC functionalities such as cell survival, migration, and proliferation [50]. In addition, the inhibition of intracellular PI3K/Akt signal cascade has been shown to increase senescence-associated changes and decrease the expression of human telomerase reverse transcriptase in BM-MSCs [51]. Understanding these molecular interactions could provide insights into the mechanisms by which PDPN influences the p16^Ink4a^/Rb pathway through the downstream effects of CD44 activation-mediated PI3K/Akt signaling, potentially revealing new therapeutic targets.

To assess the role of PDPN in MSC senescence, we initially analyzed the effect of replicative senescence on TMSCs through serial culture. Since Hayflick demonstrated cellular senescence upon serial passaging, it has been established that senescent cells are characterized by several cellular modifications during in vitro expansion, including the loss of proliferative potential and morphological abnormalities, such as a large, flat shape and high granularity [41]. In this regard, we observed increases in the doubling time, cell size, and SA-β-gal-positivity of TMSCs passaged more than 15 times. Furthermore, telomere shortening, another hallmark of replicative senescence in MSCs [52], was observed in TMSCs passaged more than 15 times, as evidenced by T/S ratio measurements. Further, replicative senescence of TMSCs reduced pluripotency-related factors, including NANOG, OCT4, and SOX2, and tri-lineage differentiation potential, as supported by previous studies [14, 53]. These findings suggest that TMSCs, after > 15 passages, can be classified as senescent cells, with their MSC properties significantly affected by senescence in vitro.

An interesting finding in our study was the significant decrease in PDPN at the protein level without a corresponding decrease at the mRNA level during replicative senescence. In particular, we observed a significant reduction in PDPN expression in both cell lysates and on the surface of senescent TMSCs. This finding aligns with previous reports indicating that proteasome-mediated degradation regulates PDPN protein expression, which is essential for osteocyte differentiation in mouse osteocyte lines [54]. This may be because the quality and abundance of membrane proteins are often regulated by ubiquitination, a multistep enzymatic process that attaches ubiquitin or ubiquitin chains to target proteins [55]. Furthermore, Cdh1 induced by stress exposure reportedly leads to SIRT1 ubiquitination and degradation, resulting in the induction of premature senescence via the p53/p21^CIP1^ pathway [56]. Although no direct evidence or mechanism has been elucidated regarding the regulation of PDPN by ubiquitination during the senescence of TMSCs, we suggest that ubiquitin-proteasome-mediated PDPN protein degradation may contribute to promoting senescence.

Additionally, we evaluated the role of PDPN in the inherent properties of MSCs, which are lost through replicative senescence. We found that PDPN^−^ cells, sorted from a heterogeneous pool of TMSCs, exhibited significantly lower tri-lineage differentiation potential than PDPN^+^ cells. However, pluripotency-related factor expression was maintained after sorting TMSCs according to the level of PDPN. Furthermore, telomere length did not differ between PDPN^+^ and PDPN^−^ cells. It has been shown that not only are the pluripotency-related factors expressed in MSCs regulated according to the duration of expansion [57], but the telomere length of MSCs is also typically regulated by cell division in replicative in vitro culture [58]. These results indicated that culture conditions after sorting PDPN may not be sufficient to induce changes in pluripotency markers and telomere length. In this regard, our results provide evidence to explain the dual role of PDPN in MSC properties and cellular senescence.

## Conclusions

This study demonstrated that PDPN contributes to the modulation of stemness- and senescence-related properties in MSCs. The abundance of PDPN is associated with higher MSC characteristics, such as tri-lineage differentiation potential. PDPN is also involved in cellular senescence by controlling cell-cycle progression via p16^Ink4a^/Rb pathway activation. Taken together, our results suggest that PDPN may serve as a biomarker for enhancing the quality of MSCs by minimizing senescence and preserving stemness. Therefore, PDPN could be used as an indicator to ensure the high quality of MSCs and MSC-based cell therapies in clinical applications and regenerative medicine.

## Data Availability

No datasets were generated or analysed during the current study.

## Abbreviations

BM-MSCs: Bone marrow-derived mesenchymal stem cells
CDK4: Cyclin-dependent kinase 4
CDKs: Cyclin-dependent kinases
DPBS: Dulbecco’s phosphate-buffered saline
ESCs: Embryonic stem cells
FACS: Fluorescence-activated cell sorting
FBS: Fetal bovine serum
FITC: Fluorescein isothiocyanate
GAPDH: Glyceraldehyde 3-phosphate dehydrogenase
ISCT: International Society for Cellular Therapy
MSCs: Mesenchymal stem cells
MTT: 3-(4,5-dimethylthiazol-2-yl)-2,5-diphenyltetrazolium bromide
PDPN: Podoplanin
PE: Phycoerythrin
PI3K: Phosphatidylinositol 3-kinase
PI: Propidium iodide
RT-PCR: Reverse transcription polymerase chain reaction
SA-β-gal: Senescence-associated β-galactosidase
siCON: Control small interfering RNA
siPDPN: PDPN small interfering RNA
siRNA: Small interfering RNA
TMSCs: Tonsil-derived mesenchymal stem cells
UC-MSCs: Umbilical cord-derived mesenchymal stem cells
